# Supplementary material for: Dominance rank but not body size influences female reproductive success in mountain gorillas
Source: PLoS One. 2020 Jun 3;15(6):e0233235. doi: 10.1371/journal.pone.0233235 (PMC7269200; doi:10.1371/journal.pone.0233235)
Supplement: S1 Material — (DOCX) [file pone.0233235.s001.docx]

Supplementary Material

*Photogrammetry error*

Measurements were based on an average of six photos per female and trait (range: 3 – 10). Within-individual error was assessed by comparing measurements from different photos of the same trait and female. Coefficients of variation (CVs) were low at around 2% (Table S1).

Table S1. Within-individual error

| Morphological trait | Mean within-individual CV % |
| --- | --- |
| Back breadth | 2.0 (0.9 – 2.8) |
| Body length | 2.2 (0.7 – 3.8) |

CV: coefficients of variation are displayed as a %. Numbers in brackets indicate minimum and maximum values. See also inter-individual error in Table 1 and [1] for further details of photogrammetry error.

*Inter-birth interval model – with all three predictors and control variables*

A likely over-parameterized model with the three predictors and four control variables (mother age, offspring sex, parity of mother and whether the mother transferred in between the birth of the two infants) did not reveal significance (non-significant full null model comparison: Χ^2^ = 6.0, *df* = 3, *p* = 0.112), likely caused by low statistical power. Yet, the individual p-value for dominance rank was significant (keeping in mind the risk of multiple testing [2; Table S2]. Higher-ranking females had significantly shorter inter-birth intervals than lower-ranking ones. Both back breadth and body length did not significantly influence inter-birth interval duration (Table S2). These results are similar to those found using univariate analyses presented in the text. The only control predictor to significantly influence inter-birth interval duration was the sex of the offspring, with intervals being longer for male rather than female offspring (Table S2).

Table S2. The influence of dominance rank, back breadth, body length and four additional control variables on inter-birth interval duration.

| Predictors | *Estimate* | *Standard error* | CI lower | CI upper | *X*^2^ | *p* |
| --- | --- | --- | --- | --- | --- | --- |
| Intercept | 41.71 | *2.79* | 35.81 | 47.32 |  |  |
| Dominance rank | -3.18 | 1.36 | -6.00 | -0.37 | 4.79 | 0.03 |
| Back breadth | -1.15 | 1.33 | -3.75 | 1.72 | 0.71 | 0.40 |
| Body length | 0.96 | 1.31 | -1.88 | 3.38 | 0.53 | 0.47 |
| Mother age | 1.49 | 1.52 | -1.73 | 4.76 | 0.87 | 0.35 |
| Offspring sex | 6.34 | 1.85 | 2.75 | 9.93 | 9.25 | 0.00 |
| Parity | 2.14 | 3.12 | -4.41 | 8.46 | 0.46 | 0.50 |
| Transfer y/n | -1.23 | 2.22 | -6.18 | 3.71 | 0.28 | 0.60 |

Quantitative predictors were z-transformed to a mean of 0 and a standard deviation of 1. The degrees of freedom for all variables was 1. CI refers to 2.5% (lower) and 97.5% (upper) confidence intervals. Values for offspring sex are in relation to male offspring, values for parity are in relation to primiparous females and values for transfer y/n are in relation to females who did not transfer in between the birth of the two infants in the inter-birth interval.

*Infant mortality model – with all three predictors and control variables*

This model was also potentially over-parameterized and a comparison of the full model comprising dominance rank, back breadth and body length with a null model with these variables excluded, comprising the control variables (mother age, parity and group size) indicated that these variables did not significantly influence infant mortality (likelihood ratio test: Χ^2^ =1.281, *df* = 3, *p* = 0.734; Table S3; similar to the results from the univariate analyses reported in the text).

Table S3. The influence of dominance rank, back breadth, body length and other variables on infant mortality.

| Predictors | *Estimate* | *Standard error* | *X* ^2^ | *p* |
| --- | --- | --- | --- | --- |
| Dominance rank | 0.13 | 0.31 | 0.19 | 0.66 |
| Back breadth | -0.37 | 0.35 | 1.07 | 0.30 |
| Body length | 0.26 | 0.33 | 0.69 | 0.41 |
| Mother age | 0.40 | 0.32 | 1.55 | 0.21 |
| Parity | 0.79 | 0.91 | 0.68 | 0.41 |
| Group size | -0.19 | 0.26 | 0.41 | 0.52 |

Quantitative predictors were z-transformed to a mean of 0 and a standard deviation of 1. The degrees of freedom for all variables was 1. Values for parity are in relation to primiparous females.
