# Supplementary material for: Dominance rank but not body size influences female reproductive success in mountain gorillas
Source: PLoS One. 2020 Jun 3;15(6):e0233235. doi: 10.1371/journal.pone.0233235 (PMC7269200; doi:10.1371/journal.pone.0233235)
Supplement: Attachment — Submitted filename: Response_to_Reviewers.docx [file pone.0233235.s006.docx]

PONE-D-19-28202R1
Dominance rank but not body size influences female reproductive success in mountain gorillas
PLOS ONE

Dear Dr. Wright,

Thank you for submitting your manuscript to PLOS ONE. After careful consideration, we feel that it has merit but does not fully meet PLOS ONE’s publication criteria as it currently stands. Therefore, we invite you to submit a revised version of the manuscript that addresses the points raised during the review process.

Both reviewers and myself found that you dealt adequately with the previous comments and resubmit a stronger version of the manuscript. However, both reviewers have some minor suggestions to further improve the clarity of the manuscript.

We would appreciate receiving your revised manuscript by Apr 12 2020 11:59PM. When you are ready to submit your revision, log on to <https://www.editorialmanager.com/pone/> and select the 'Submissions Needing Revision' folder to locate your manuscript file.

If you would like to make changes to your financial disclosure, please include your updated statement in your cover letter.

To enhance the reproducibility of your results, we recommend that if applicable you deposit your laboratory protocols in protocols.io, where a protocol can be assigned its own identifier (DOI) such that it can be cited independently in the future. For instructions see: <http://journals.plos.org/plosone/s/submission-guidelines#loc-laboratory-protocols>

Please include the following items when submitting your revised manuscript:

- A rebuttal letter that responds to each point raised by the academic editor and reviewer(s). This letter should be uploaded as separate file and labeled 'Response to Reviewers'.
- A marked-up copy of your manuscript that highlights changes made to the original version. This file should be uploaded as separate file and labeled 'Revised Manuscript with Track Changes'.
- An unmarked version of your revised paper without tracked changes. This file should be uploaded as separate file and labeled 'Manuscript'.

Please note while forming your response, if your article is accepted, you may have the opportunity to make the peer review history publicly available. The record will include editor decision letters (with reviews) and your responses to reviewer comments. If eligible, we will contact you to opt in or out.

We look forward to receiving your revised manuscript.

Kind regards,

Julien Martin
Academic Editor
PLOS ONE

Dear Julien,

We are happy to hear that both reviewers and you were happy with the changes we made and with the revised manuscript. We have now carefully gone through these additional minor comments and hope that you will now be satisfied with the current version.

Many thanks.

Kind regards,

Edward Wright

[Note: HTML markup is below. Please do not edit.]

Reviewers' comments:

Reviewer's Responses to Questions

**Comments to the Author**

1. If the authors have adequately addressed your comments raised in a previous round of review and you feel that this manuscript is now acceptable for publication, you may indicate that here to bypass the “Comments to the Author” section, enter your conflict of interest statement in the “Confidential to Editor” section, and submit your "Accept" recommendation.

Reviewer #1: (No Response)

Reviewer #2: (No Response)

2. Is the manuscript technically sound, and do the data support the conclusions?

The manuscript must describe a technically sound piece of scientific research with data that supports the conclusions. Experiments must have been conducted rigorously, with appropriate controls, replication, and sample sizes. The conclusions must be drawn appropriately based on the data presented.

Reviewer #1: Yes

Reviewer #2: Yes

3. Has the statistical analysis been performed appropriately and rigorously?

Reviewer #1: Yes

Reviewer #2: Yes

4. Have the authors made all data underlying the findings in their manuscript fully available?

The [PLOS Data policy](http://www.plosone.org/static/policies.action#sharing) requires authors to make all data underlying the findings described in their manuscript fully available without restriction, with rare exception (please refer to the Data Availability Statement in the manuscript PDF file). The data should be provided as part of the manuscript or its supporting information, or deposited to a public repository. For example, in addition to summary statistics, the data points behind means, medians and variance measures should be available. If there are restrictions on publicly sharing data—e.g. participant privacy or use of data from a third party—those must be specified.

Reviewer #1: Yes

Reviewer #2: Yes

5. Is the manuscript presented in an intelligible fashion and written in standard English?

PLOS ONE does not copyedit accepted manuscripts, so the language in submitted articles must be clear, correct, and unambiguous. Any typographical or grammatical errors should be corrected at revision, so please note any specific errors here.

Reviewer #1: Yes

Reviewer #2: Yes

6. Review Comments to the Author

Please use the space provided to explain your answers to the questions above. You may also include additional comments for the author, including concerns about dual publication, research ethics, or publication ethics. (Please upload your review as an attachment if it exceeds 20,000 characters)

Reviewer #1: Thank you for your thoughtful editing of this manuscript. I have a few smaller comments remaining. I think all major issues have been addressed.

We were pleased to hear that you were much happier with the revised manuscript. We have now carefully gone through these additional comments and we hope that you will now be fully satisfied with the current version. We thank you once again for your efforts in helping us improve this manuscript.

Abstract:

The sentence on lines 22-26 is very long and the ideas aren’t connected in a way that’s easy to understand. Creating two sentences and clarifying would do the trick. I think you’re saying that (1) Previous studies saw a relationship between rank and reproduction, (2) that’s surprising because their hierarchy is relatively weak, (3) maybe hierarchy is weak because feeding competition is weak, (4) maybe rank is associated with body size because...[of the association between rank and reproduction?]

We agree that this sentence could be improved and we have now rephrased it as follows:

Previous studies found that female dominance rank correlates with reproductive success in mountain gorillas (G*orilla beringei beringei*), which is surprising given they have weak dominance relationships and experience seemingly low levels of feeding competition. It is not currently known whether this relationship is primarily driven by a positive correlation between rank and body size.

Line 31: You say ‘no support for body size influencing…’ but I don’t think you can assume causation. The word ‘influence’ is used in line 34 as well.

We agree that we cannot assume causation here and we have now replaced “influence” with “correlation”. The two new sentences are as follows:

“Using linear mixed models, we found no support for body size to be significantly correlated with dominance rank or female reproductive success. Higher-ranking females had significantly shorter inter-birth intervals than lower-ranking ones, but dominance rank was not significantly correlated with infant mortality.”

Introduction:

The first four paragraphs don’t connect or ‘flow’ together well, so it is challenging to read. Perhaps the first sentence of each paragraphs 2-4 should include a transition from the previous paragraph’s topic to the current paragraph’s topic to help the reader.

We have rewritten the first sentences of paragraphs 3, 4, and 5 to improve the transitions among paragraphs and improve the readability of these paragraphs. We realize we are covering a lot of ground in these few paragraphs and the linkages may not be so obvious to all readers. Thank you for pointing this out.

Line 66-67: Thanks for shifting this sentence around. One more suggestion is that ‘optimal’ isn’t necessarily right. Not only is selection acting on the whole organism (not just body size), but selection isn’t an optimizing process.

We have now replaced the word “optimal’ with “ideal”.

Line 121-122: If you shift the parenthetical to the end of the sentence it will be easier to read.

We have now placed the parenthesis at the end of the sentence.

Line 124: It’s not yet clear why the authors thought the rank-reproduction association might be attributable to a rank-body size association – spelling it out clearly will help lead the reader to your present study

We have now rephrased this sentence and we think it is now clearer to understand:

Given the low levels of feeding competition and weak dominance relationships, such relationships were not expected and those authors suggested that the positive correlation between dominance rank and reproductive success may in fact be a by-product of a positive correlation between rank and body size, such that body size is driving the relationship, not rank.

Line 134: You already know that rank is associated with these reproductive measures in this population, right? If so, perhaps include that as a citation and explain why you’re testing it again.

We are testing it again because we have a different data set and we believe it is useful to also test if the relationship between rank and reproductive success still holds in this population, especially since it was not expected.

Results:

Line 283: It’s helpful for the reader to briefly give the formula for %CV for those unfamiliar with it.

We have now included the formula for %CV in this table.

Thanks for adding in the CVs for each individual (Table S1). I didn’t see that table directly referenced in the text; perhaps it could be referenced in the caption for Table 1 (and sorry if I missed it elsewhere).

Thank you for highlighting this. We have now referenced Table S1 in the caption of Table 1 to refer readers to intra-individual CVs.

Line 317: Given your confidence interval for back breadth, I don’t think it’s helpful to mention this last sentence.

We agree, and we have now deleted this sentence.

Discussion:

Line 385: There’s an extra comma after “Even though”

We have deleted this comma, thank you.

Line 418: Specify whether the rank-reproduction relationship in chimpanzees is in females specifically.

We now make it clear that this relationship is present in females specifically.

Reviewer #2: I would like to praise the authors for their constructive responses and for the changes they have made following the comments from both revisions. Although I am normally more in favor of an information-theoretic approach, data do not always justify or allow its use. Since it was repeated multiple times in the authors’ response to reviewers that the sample size had low power, I run a couple of power analyses myself to test what was the power given by the available dataset and different R2 values. I thus agree that the statistical approach used here is valid and justified by the nature and amount of available data. I appreciate the improved clarity on the choice of univariate vs multivariate models.
I highlight here a few possible minor changes, but I otherwise think this article is a sound and fascinating work that will be received with interested by the ecological community.

Best wishes,
Luca Montana

We are happy to hear that you were satisfied with our changes and that you agree with our statistical approach. Thank you for your additional suggestions. We have now carefully gone through these and made the appropriate changes. Thank you once again for your efforts in helping to improve this manuscript.

Minor comments
1. Ll 66-67. I would suggest to use the present tense, so “[…] selection shapes individuals to be an optimal size to maximize reproductive success”. In its present form, it seems like individuals already attained the perfect size to maximise their fitness. If that was the case, the variance on a trait such as body size would be greatly reduced.

Thank you for this suggestion. We have now changed this sentence to the present tense and replaced the word ‘optimal’.

2. Ll. 230-232. I think it would be helpful to disclose the link function of the beta model used to test the first hypothesis. I assume it was a logit function?

Yes, you are correct it is a logit function which we now specify in the text.

3. Ll 275-276. Should this paragraph be included in a specific section like Ethical statement or something like it?

We have now added “Ethical Note” above this paragraph.

4. Ll 329-330. Is there any reference that would support this assumption? Probably Grueter et al. (2016), Wright and Robbins (2014) or one among the references cited in l 379? This is merely because if somebody jumps directly to the discussion the absence of references could suggest a lack of knowledge to sustain this, although logical, assumption.

Thank you for this suggestion. We have now added both Grueter et al. 2016 and Wright et al. 2014 citations to this sentence.

5. Ll 384-388. I wonder if the comma after ‘even though’ in the following sentence is appropriate “Even though, higher-ranking females had greater access to some food resources over lower-ranking ones, dominance rank did not significantly predict energy intake rate or levels of urinary C-peptide, a common proxy for energy balance”. I am not totally sure since I’m not a native english speaker,

We have now deleted this comma.

6. Ll. 435-441. I wonder if this last paragraph is relevant to the discussion. Sexual size dimorphism is only briefly cited in the introduction, and given that selection on body size was not formally tested here I would suggest to remove it entirely.

Thank you for this suggestion to streamline this section of the discussion. Whilst we agree that sexual size dimorphism does not form a central part of the study, we still think it is a useful short section to include for two reasons. 1) we do provide and discuss the equivalent data on male gorillas for comparative value and 2) this section helps to better understand why females are considerably smaller than males overall. As this is a small section we do not think it distracts the readers from the main message of the study.

7. Supplementary material. I think that the appropriate reference for this sentence between parentheses “keeping in mind the risk of multiple testing” was actually 2 (Forstmeier and Schielzeth 2011) and not 1 (Wright et al. 2019).

You are right, thank you very much for highlighting this mistake.

References
Forstmeier W, Schielzeth H. 2011. Cryptic multiple hypotheses testing in linear models: Overestimated effect sizes and the winner’s curse. Behav Ecol Sociobiol 65:47–55. doi:10.1007/s00265-010-1038-5.
Grueter CC, Robbins AM, Abavandimwe D, Vecellio V, Ndagijimana F, Ortmann S, Stoinski TS, Robbins MM. 2016. Causes, mechanisms, and consequences of contest competition among female mountain gorillas in Rwanda. Behav Ecol 27:766–776. doi:10.1093/beheco/arv212.
Wright E, Galbany J, McFarlin SC, Ndayishimiye E, Stoinski TS, Robbins MM. 2019. Male body size, dominance rank and strategic use of aggression in a group-living mammal. Anim Behav 151:87–102. doi:10.1016/j.anbehav.2019.03.011.
Wright E, Robbins MM. 2014. Proximate mechanisms of contest competition among female Bwindi mountain gorillas (Gorilla beringei beringei). Behav Ecol Sociobiol 68:1785–1797. doi:10.1007/s00265-014-1788-6.

7. PLOS authors have the option to publish the peer review history of their article ([what does this mean?](https://journals.plos.org/plosone/s/editorial-and-peer-review-process#loc-peer-review-history)). If published, this will include your full peer review and any attached files.


If you choose “no”, your identity will remain anonymous but your review may still be made public.


**Do you want your identity to be public for this peer review?** For information about this choice, including consent withdrawal, please see our [Privacy Policy](https://www.plos.org/privacy-policy).

Reviewer #1: Yes: Emily Levy

Reviewer #2: Yes: Luca Montana


[NOTE: If reviewer comments were submitted as an attachment file, they will be attached to this email and accessible via the submission site. Please log into your account, locate the manuscript record, and check for the action link "View Attachments". If this link does not appear, there are no attachment files to be viewed.]


While revising your submission, please upload your figure files to the Preflight Analysis and Conversion Engine (PACE) digital diagnostic tool, <https://pacev2.apexcovantage.com/>. PACE helps ensure that figures meet PLOS requirements. To use PACE, you must first register as a user. Registration is free. Then, login and navigate to the UPLOAD tab, where you will find detailed instructions on how to use the tool. If you encounter any issues or have any questions when using PACE, please email us at [figures@plos.org](mailto:figures@plos.org). Please note that Supporting Information files do not need this step.

*In compliance with data protection regulations, you may request that we remove your personal registration details at any time.* [*(Remove my information/details)*](https://www.editorialmanager.com/pone/login.asp?a=r)*. Please contact the publication office if you have any questions.*
